# Discussion on Vaccination

**Published:** 1892-11

**Authors:** 


*Second Day—Afternoon Session.*

WEDNESDAY, June 22d, 4 P. M.

DISCUSSION ON VACCINATION.

Dr. J. V. Allen—I am one of those who believe that vaccination is a detriment to the health, and I want to express myself in regard to vaccinating children on going to the public schools, the children being brought to me by the parents for the purpose of gaining admission to the schools. For this purpose I use a preparation of the crystals of Antimonium-tartaricum. I tell the parents that I believe vaccination to be a bad thing, as ordinarily practiced, and that therefore I will use a preparation of Tartar-emetic. I scratch the child's arm, moisten it with the solution, and let them go. If they insist on the usual vaccination, they have to go somewhere else for it. The preparation is simply a saturated solution of Tartar-emetic. The effect is to make a few pustules on the body with perhaps slight fever, but no scar is left. I think it would be an excellent procedure in an epidemic of small-pox.

Dr. Reed—These two papers are a credit to the society and to the authors. In thirty years' practice I have seen a good deal of the evil effects of vaccination. In Macon, Missouri, there were two ladies vaccinated by a physician. In both of those ladies a serious case of eczema broke out on the arm on which they were vaccinated; fever came with it; then it spread to the lower extremities. One of the ladies died of that condition, and the other was referred to me. The leg was in a terrible plight, she could not bear bandages on it; the fluid that exuded from the sores was so excoriating that it produced sores in other parts that it came in contact with. The itching was intense, the skin exfoliated in large patches. I have several of those flakes of skin at my office. I sent her one dose of Pulsatilla. Next morning she traveled to my office, one hundred and forty-four miles. In four days' time it required repetition. In two days more it required a second repetition. Then Lycopodium was given, followed by wonderful improvement, lasting six days; then a repetition, the effect of which lasted four days. I then wrote a letter to Dr. Lippe describing the case. He wrote back that I should give Thuja. I did not consider it indicated, but gave it as he directed, without any good result whatever, but there was a residue of symptoms there which Lycopodium would not relieve. I took a flannel bandage and bandaged the limb from toe to hip, and renewed it twice a day, morning and evening. It got well and she has never been sick since.

Another terrible case of the results of vaccination comes to mind. I was asked to go down to see a woman who was said to be dying, and by the time I got there she had passed from time to eternity. As she lay on the bed I estimated the weight of the body to be three hundred pounds. It was a case of elephantiasis. Fifteen years previous to her death she had been vaccinated, and that was the beginning of the disease. Her mother, her father, and her sister had all been vaccinated at the same time, by the same physician, and they all of them had elephantiasis since that unfortunate inoculation. The thigh of this woman was as large as a large man's body ; it was split in several places and a fluid exuded. Before the vaccination this family had been free from chronic disease of any kind.

Dr. Rushmore—Do you know anything of the source of the lymph?

Dr. Reed—No, sir. I could find out that it was animal virus, but nothing more.

Dr. Baylies—I know of two young girls who tell me they were vaccinated at the same time, more than two years ago, with the same lymph, one of whom has suffered ever since from an ugly eruption on the face. It is red, tubercular, and papular eruption, covering the whole face, aggravated at the menstrual period. Thuja has much relieved but not entirely cured her. It may in time.

Dr. J. H. Allen—The effect of vaccination is, I believe, a from of sycosis, and that is the reason that Thuja so frequently cures it.

Dr. S. Close—Perhaps I may say a few words as to the practical application of these principles. It has been my custom to give Variolinum$^{900}$, to children applying to me for vaccination. This meets, as far as I know, the requirements of the local authorities in Brooklyn. At the end of the time usually devoted to observation of the case, I give a certificate stating that the child has been vaccinated homœopathically. If any contest comes up in regard to this, it would involve our right to prescribe homœopathically. It is our privilege to produce proplylaxis, as well as to treat the sick, by homœopathic methods, and this is our method of vaccinating.

As to what effects are produced, in some cases no effect; in other cases symptoms are produced, the most prominent being slight headache in occiput down to spine, backache and elevation of the temperature, restlessness, and, in a few cases, a slight eruption on the skin, chiefly on the forehead, neck, and back of hands. I usually give three powders, one to be taken each successive night. I suppose I have given twenty-five to fifty certificates. I decline to vaccinate by the ordinary methods. When parents come to me, I briefly explain my position, and, if they disagree, I put in their hands some literature on the subject.

Dr. Baylies—The following case accords with a proving of Variolinum given me by Dr. Fincke. Preston F——, aged five years, and sister about seven years of age, each took three powders of Variolinum$^{900}$, Fincke, as follows: One powder dry on the tongue night and morning, on the 24th and 25th of April, 1891. On May 1st, the seventh day following the doses, an eruption appeared on Preston's face and neck, and quickly spread over his body. "Some of the blotches," wrote his father, "were as large as a silver five-cent piece, and held a watery pus," which gradually dried up, leaving a yellowish scale. "He had fever on the night of May 1st, continuing till it subsided somewhat on May 3d, and he was then feeling better." May 13th. "The eruption had about disappeared." No symptoms were remarked upon the sister.

Dr. Hitchcock—I consider this a question of extreme importance to the community. I have therefore given the legal aspects of the case. In relation to the effects that vaccination produces, I should like to read the record of one case which is very horrible, and which shows that vaccination laws are murderous.

